# Maternal Conjugated Linoleic Acid Supply in Combination With or Without Essential Fatty Acids During Late Pregnancy and Early Lactation: Investigations on Physico-Chemical Characteristics of the Jejunal Content and Jejunal Microbiota in Neonatal Calves

**DOI:** 10.3389/fvets.2022.839860

**Published:** 2022-03-25

**Authors:** Wendy Liermann, Katrin Lena Wissing, Henry Reyer, Nares Trakooljul, Dirk Dannenberger, Arnulf Tröscher, Harald Michael Hammon

**Affiliations:** ^1^Institute of Nutritional Physiology “Oskar Kellner”, Research Institute for Farm Animal Biology (FBN), Dummerstorf, Germany; ^2^Institute of Genome Biology, Research Institute for Farm Animal Biology (FBN), Dummerstorf, Germany; ^3^Institute of Muscle Biology and Growth, Research Institute for Farm Animal Biology (FBN), Dummerstorf, Germany; ^4^BASF SE, Lampertheim, Germany

**Keywords:** conjugated linoleic acid, calf, small intestine, fatty acids, microbiota

## Abstract

Conjugated linoleic acids (CLAs) modulate the fatty acid composition in dairy cow milk, which represents the most important nutrient source of neonatal calves. In turn, dietary fatty acids are known to influence the gut microbiota. The current preliminary study investigated effects of a maternal fatty acid supplementation (MFAS) during transition period with coconut oil (CON, control), CLA (Lutalin®), or CLA + EFA (Lutalin® + essential fatty acids–linseed oil; safflower oil) on physico-chemical characteristics of jejunal content and microbiota of 5-day-old calves. MFAS of CLA + EFA increased α-linolenic, eicosapentaenoic, docosapentaenoic, and n-3 fatty acid proportions in jejunum compared to the other groups (*P* < 0.05). Proportions of n-6 and polyunsaturated fatty acids increased by MFAS of CLA + EFA compared to CON (*P* < 0.05). Most abundant phyla in the jejunum were *Proteobacteria, Firmicutes*, and *Bacteroidota*. CLA + EFA decreased the relative abundance of *Diplorickettsiales* (*Proteobacteria*) compared to CON and CLA (*P* < 0.05). CLA calves showed a lower abundance of *Enterobacterales* (*Proteobacteria*) compared to CON calves (*P* = 0.001). The abundance of *Veillonellales-Selenomonadales* and *RF39* (*Firmicutes*) decreased in CLA + EFA calves compared to CON calves (*P* < 0.05). *Bacteroidales* (*Bacteroidota*) decreased in CLA + EFA calves compared to CLA calves (*P* < 0.05). The relative abundance of *Cyanobacteria* and *Euryarchaeota* decreased and the abundance of *Chloroflexi* increased in CLA + EFA calves compared to CON and CLA calves (*P* < 0.05). MFAS alters the fatty acid composition and microbial milieu in the intestinal content of neonatal calves due to their ability to modulate colostral fatty acid composition of dams.

## Introduction

Conjugated linoleic acid (CLA) isomers occur naturally in dairy products (1). The most abundant and bioactive isomers are cis-9, trans-11 and trans-10, cis-12 CLA (1). CLA isomers are mainly synthesized by ruminal bacteria in the forestomach depending on the intake of linoleic and α-linolenic acid, which are representatives of essential fatty acids (EFAs) and precursors for the CLA synthesis (1–3). Haubold et al. (4) and Vogel et al. (5) demonstrated the ability of CLA to influence the chemical and fatty acid composition of colostrum and milk. In the 1st days of life, the colostrum and transition milk are the most important nutrient sources of calves (6). Besides beneficial immunologic components such as antimicrobial proteins, immunoglobulins, cytokines, growth factors, and immune cells, the colostrum also contains a wide range of microbes such as *Lactobacilli* or *Bifidobacteria* (7). Therefore, the colostrum plays an important role in the development of the gastrointestinal microbiota of the offspring (7). In turn, fatty acids are also known to modulate the composition of the gut microbiota (8). A direct supplementation of trans-10, cis-12 CLA altered the cecal concentrations of short-chain fatty acids and the cecal microbial composition in studies of mice (9). It was also shown that the maternal fatty acid status in mice has a crucial impact on the microbiota of the offspring (10). Because the early intestinal microbiota plays a crucial role in direct and long-term health of neonates (10–12), the aim of the present study was to investigate the effects of a maternal CLA supplementation with or without an additional EFA supply on physico-chemical characteristics of the intestinal content and the microbial composition. It was hypothesized that the maternal CLA supplementation influences the fatty acid composition of the intestinal content and has beneficial effects on the intestinal microbiota in neonatal calves, and an additional EFA supplementation might intensify the effects of CLA.

## Materials and Methods

The current experiment was performed in accordance with the guidelines of the German Animal Protection Law at the experimental station of the Research Institute for Farm Animal Biology (FBN), Dummerstorf. The housing conditions and experimental procedures were approved by the Landesamt für Landwirtschaft, Lebensmittelsicherheit, und Fischerei Mecklenburg-Vorpommern, Rostock (registration number 7221.3-1-052/15).

### Experimental Design and Animals

Nine Holstein cows (second lactation) were abomasally supplemented *via* rumen fistula either with coconut oil (76 g/day) as a saturated fatty acid source and providing no α-linolenic acid and CLA (control, CON; Bio-Kokosöl #665, Kräuterhaus Sanct Bernhard KG, Bad Ditzenbach, Germany) or Lutalin® (38 g/day) [CLA; corresponding to cis-9 (10 g/day), trans-11, trans-10 (10 g/day), cis-12 CLA; BASF SE, Ludwigshafen, Germany] from 63 days before until day 5 after calving (5). A third group was supplemented with a combination of CLA + EFA including Lutalin® (38 g/day), linseed oil (78 g/day), and safflower oil (4 g/day) as EFA sources (DERBY® Leinöl #4026921003087, DERBY® Spezialfutter GmbH, Münster, Germany; GEFRO® Distelöl, GEFRO® Reformversand Frommlet KG, Memmingen, Germany). Each dose was halved during dry period beginning 6 weeks before expected calving. Doses for the supplied CLA and EFA (linseed and safflower oil in a ratio of 19.5:1; providing an n-6/n-3 fatty acid ratio of 1:3 in the supplemented mixture) were evaluated in a companion dose-response study by Haubold et al. (4). The basal diet was a corn silage–based total mixed ratio as described by Vogel et al. (5).

Calves born from corresponding cows (CON, *n* = 3 and 2 male calves and 1 female calf; CLA, *n* = 3 and 1 male calves and 2 female calves; CLA + EFA, *n* = 3 and 2 male calves and 1 female calf) were separated from their dams immediately after birth. They were individually housed in boxes (1.4 × 2.45 m), which were integrated in a climate-controlled room and littered with straw. The constant ambient temperature was 19°C. Within the first 3 h after birth, calves were supplied with first colostrum from respective dams. On day 1 after birth, calves were fed colostrum from respective dams at an amount of 10% of body weight provided in two meals. On day 2 after birth, only 6% colostrum of body weight was fed to ensure a similar intake within the first 48 h for all calves. From day 3 on, transition milk of respective dams gained in the morning was provided at an amount of 12% of body weight, split into two meals. In case of insufficiency of the individual morning milking, the milk of the second milking of the respective day and dam was used. If both milkings were insufficient, then the colostrum/transition milk of a dam from the respective group and milking was added. However, this procedure had to be used only in case of one calf. In general, calves were fed by nipple bottle but refused colostrum was tube-fed. Water was available *ad libitum* by a water trough.

### Sampling Procedures and Analyses of Intestinal Content

On day 5 after birth, calves were slaughtered by bolt shooting and exsanguination 2 h after feeding. A representative sample of the intestinal content was collected from the mid-jejunum in 50-ml tubes. The pH of intestinal content was determined immediately after extraction. Samples were frozen at −20°C in 50 ml of aliquots until further analyses.

Aliquots were thawed gently. Dry matter and moisture content were determined by an automatic moisture analyzer (Satorius Moisture Analyzer MA37, Satorius Lab Instruments, Göttingen, Germany).

For fatty acid composition analyses, an aliquot of the intestinal content was freeze-dried and homogenized. Subsequently, samples were prepared and fatty acid composition was measured according to the methods described by Dannenberger et al. (13, 14).

For the determination of lactate and volatile fatty acids (VFA; acetic acid, iso-butyric acid, butyric acid, propionic acid, and iso-valeric and valeric acid), an aliquot of the intestinal content was centrifuged at 3,000 × g and room temperature, initially. The supernatant was divided in aliquots. Lactate was determined colorimetrically according to methods of Haacker et al. (15). VFA concentrations were measured by gas chromatography. Sample preparation was similar to methods described by Tümmler et al. (16). The measurements were conducted using a gas chromatograph (GC-2010 Plus, Shimadzu Corp., Kyoto, Japan) equipped with a flame ionization detector and a 25 × 0.25-mm free fatty acid phase column (Roth, Karlsruhe, Germany).

### Isolation of Microbial DNA

The microbial DNA was isolated using the QIAmp® DNA Stool Kit (QIAGEN, Hilden, Germany). Thus, ~400 μg of semi-frozen mixed intestinal content was transferred in a 2-ml PowerBead Tube (glass, 0.1 mm; QIAGEN). ASL Buffer (1 ml) (provided by the kit) was added. The samples were homogenized by an automatic homogenizer (Precellys® Evolution, Bertin Technologies, Montigny le Bretonneux, France) for 45 s at 4,500 rounds per minute (RPM) twice including an intermediated resting phase of 30 s. An incubation period of 5 min at 95°C followed. Thereafter, samples were vortexed for 15 s and centrifuged at 16,000 × g for 1 min and room temperature. One ml of the supernatant was transferred into a 2-ml tube. The pellet was rejected. One InhibitEx pill was added to the supernatant. The samples were vortexed until the pill was completely dissolved. After a resting phase of 1 min, the samples were centrifuged for 3 min at 16,000 × g. The supernatant was transferred in a new 1.5-ml tube and centrifuged for 3 min at 16,000 × g once again. A total of 200 μl of the supernatant was transferred in a 1.5-ml tube containing 15 μl of proteinase K and 200 μl of AL Buffer (provided by the QIAmp® DNA Stool Kit). The samples were vortexed for 15 s and incubated for 10 min at 70°C subsequently. The samples were mixed with 200 μl of ethanol and thereafter transferred on a column that was equipped with a collection tube and centrifuged for 1 min at 16,000 × g. The column was washed with 500 μl of AW1 Buffer in the first step and in a second step with 500 μl of AW2 Buffer (both buffers prepared by the kit). The filtrate was rejected in all steps. The column was transferred in a new collection tube and centrifuged for 1 min at 16,000 × g once again to remove rests of buffers. Thereafter, the column was equipped with a 1.5-ml tube, and 50 μl of AE Buffer (provided by the QIAmp® DNA Stool Kit) was added to the column. An incubation phase of 1 min followed. The column-tube combination was centrifuged for 1 min at 16,000 × g. The concentration and quality of the DNA was measured by NanoPhotometer^TM^ UV/VIS (Implen GmbH, Munich, Germany).

### Microbial Profiling Using 16S rRNA Sequencing

The gained DNA was used to amplify PCR-fragments V4 of the hypervariable region of 16S rRNA gene targeting the 515'F (GTGBCAGCMGCCGCGGTAA) and 806R (GGACTACHVGGGTWTCTAAT) sequences (17).

The corresponding primers were designed by combining 16S-specific sequences with adapter sequences of the Illumina flow cell and specific index sequences according to the approach described by Kozich et al. (18). The PCR was performed in duplicate using the 5 Prime HotMasterMix (5 Prime, Hamburg, Germany). The protocol was composed as follows: An initial denaturation at 95°C for 2 min, followed by 30 cycles at 95°C for 30 s, 55°C for 60 s, and 72°C for 90 s, and a final extension for 10 min at 72°C. The replicates were pooled, processed over the SequalPrep Normalization Plate (Thermo Fisher Scientific, Darmstadt, Germany), and the resulting products were mixed in the same proportions.

The DNA libraries were parallel sequenced for 2 × 250–base pair paired-end reads using the HiSeq PE Rapid Cluster Kit v2 and HiSeq Rapid SBS Kit v2 on the rapid-run mode of the HiSeq 2500 system (Illumina, San Diego, CA). Sequence data were de-multiplexed and converted into FASTQ files using the bcl2fastq2 conversion software, v2.19 (Illumina). The sequence reads were processed following the mothur pipeline [version 1.37.4; Schloss et al. (19)] including references from the Silva database (release 138; https://www.arb-silva.de/). Operational taxonomic units were derived from sequences clustered by sequence identity of ≥97 % and were subsequently annotated using the Silva database at the order, class, and phylum levels.

### Statistical Analyses and Calculations

Statistical analyses were conducted using the SAS Enterprise Guide 8.1 (SAS Institute Inc., Cary, USA). The data were analyzed by variance analyses (ANOVA) using the MIXED procedure. The ANOVA model included the fixed factors maternal fatty acid supplementation (MFAS; CON, CLA, and CLA + EFA) and sex of calves (male and female). Least-squares means (LSM) and corresponding standard errors were calculated for each fixed factor. Pair-wise differences between LSM were tested by the Tukey–Kramer procedure. Differences were declared as significant if *P* < 0.05. Data of intestinal microbiota were analyzed with DESeq2 (20). For this purpose, taxa with very low abundance were excluded, leaving only those that occurred at least 10 times in at least half of the samples. The statistical analysis included the MFAS as a fixed effect and employed the negative binomial Wald test. Differences were considered significant at a Benjamini–Hochberg adjusted *P* < 0.05.

## Results

**Figure 1:**
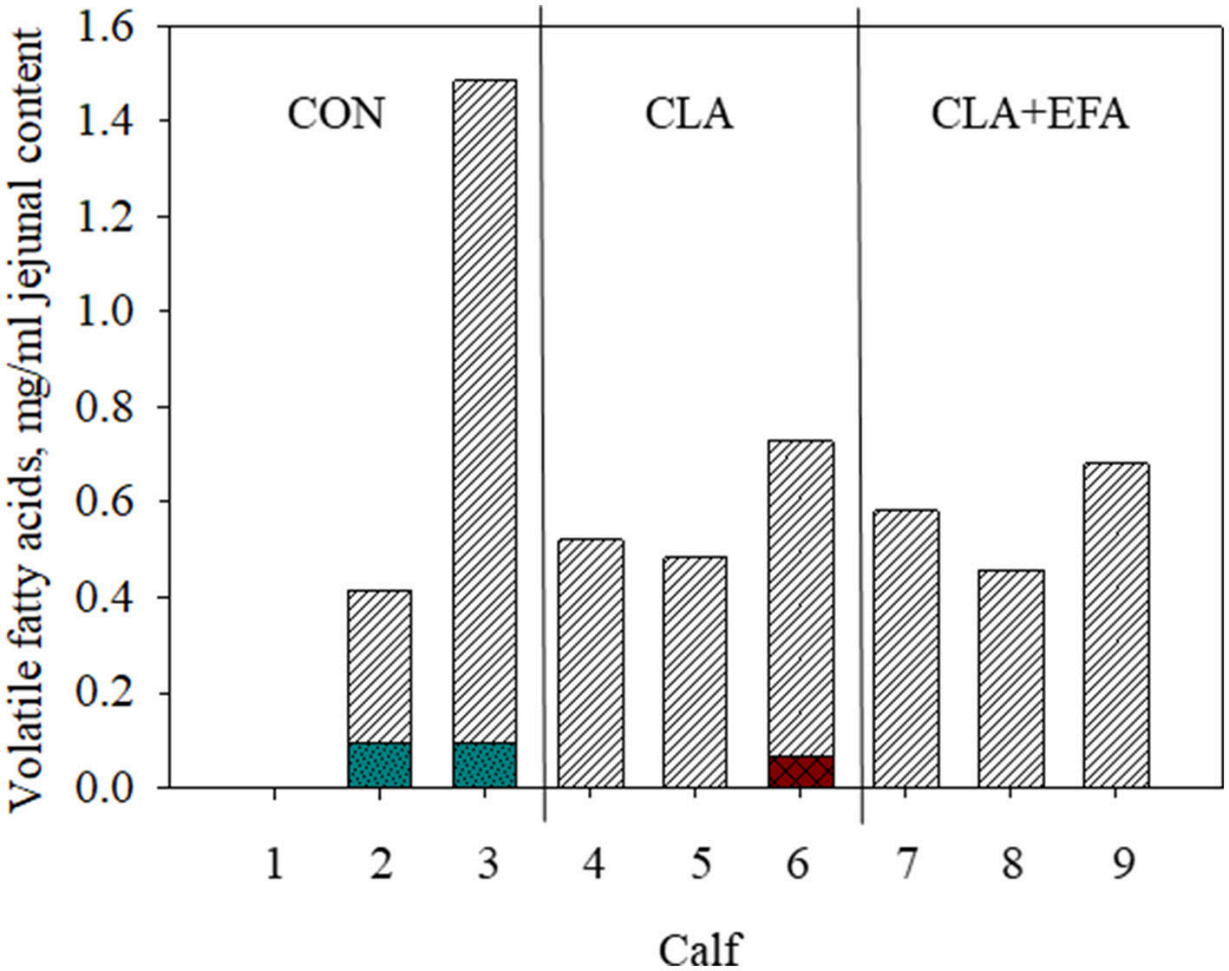
Volatile fatty acid content (striped white bars, acetic acid; checkered red bars, iso-butyric acid; pointed turquoise bars, butyric acid) in jejunal content depending on maternal fatty acid supply (CON, control group = coconut oil; CLA, conjugated linoleic acid = Lutalin® CLA + EFA = Lutalin® + linseed oil + safflower oil) in calves 1–9 (animal 1 have to be excluded from measurements because of insufficient intestinal content).

Dry matter, pH value, and concentration of lactate in jejunal chyme were not affected by MFAS (*P* > 0.05) (Table 1). Propionic, iso-valeric, and valeric acid were not detected in the jejunum of calves (Figure 1). There were no differences between concentrations of acetic acid in jejunum of calves from different groups (*P* > 0.05). Iso-butyric acid was only detected in one calf belonging to the CLA group and butyric acid only in the jejunum of CON calves.

**Table 1:** Physico-chemical characteristics of jejunal content depending on maternal fatty acid supply (MFAS)^1^ (LSM ± SE).

**Variable**	**MFAS**			**Sex**		***P*** **-values**	
	**CON** **(*n* = 3)**	**CLA** **(*n* = 3)**	**CLA + EFA** **(*n* = 3)**	**Male** **(*n* = 5)**	**Female** **(*n* = 4)**	**MFAS**	**Sex**
Dry matter, %	7.01 ± 2.29	8.56 ± 1.64	9.03 ± 1.64	7.76 ± 1.27	8.64 ± 1.88	0.76	0.73
pH	6.24 ± 0.12	6.11 ± 0.12	6.11 ± 0.12	6.11 ± 0.09	6.20 ± 0.10	0.68	0.54
Lactate, g/l	0.47 ± 0.13	0.86 ± 0.13	0.72 ± 0.13	0.84 ± 0.10	0.53 ± 0.11	0.19	0.097
**Fatty acids, %^2^**							
Linoleic acid	4.8 ± 1.8^b^	10.6 ± 1.3^ab^	13.9 ± 1.3^a^	11.0 ± 1.0	8.5 ± 1.5	0.032	0.266
α-linolenic acid	0.31 ± 0.08^b^	0.50 ± 0.06^b^	3.58 ± 0.06^a^	1.49 ± 0.05	1.43 ± 0.07	<0.001	0.50
cis-9, trans-11 CLA	0.38 ± 0.13	0.65 ± 0.09	0.67 ± 0.09	0.58 ± 0.07	0.56 ± 0.11	0.25	0.92
Arachidonic acid	0.72 ± 0.41	2.24 ± 0.29	2.56 ± 0.29	2.15 ± 0.23	1.53 ± 0.33	0.55	0.11
EPA^3^	0.09 ± 0.10^b^	0.16 ± 0.07^b^	0.70 ± 0.07^a^	0.29 ± 0.06	0.34 ± 0.08	0.007	0.71
DPA^4^	0.11 ± 0.05^b^	0.24 ± 0.04^b^	0.66 ± 0.04^a^	0.37 ± 0.03	0.31 ± 0.04	0.001	0.33
DHA^5^	0.04 ± 0.02^b^	0.08 ± 0.02^b^	0.18 ± 0.02^a^	0.11 ± 0.01	0.09 ± 0.02	0.013	0.63
Sum of n-3^6^	0.53 ± 0.18^b^	1.33 ± 0.13^b^	5.27 ± 0.13^a^	2.47 ± 0.10	2.28 ± 0.15	<0.001	0.37
Sum of n-6^7^	5.9 ± 2.4^b^	13.9 ± 1.7^ab^	17.7 ± 1.7^a^	14.3 ± 1.3	10.7 ± 2.0	0.032	0.22
Sum of SFA^8^	60.4 ± 5.8	55.3 ± 4.2	51.9 ± 4.2	52.1 ± 3.3	59.6 ± 4.8	0.51	0.29
Sum of MUFA^9^	32.7 ± 3.8	28.7 ± 2.7	24.3 ± 2.7	30.4 ± 2.1	26.7 ± 3.1	0.25	0.40
Sum of PUFA^10^	7.0 ± 2.5^b^	16.0 ± 1.8^ab^	23.7 ± 1.8^a^	17.5 ± 1.4	13.7 ± 2.0	0.01	0.22

*^1^CON, Control Group = Coconut oil. CLA, Conjugated Linoleic Acid = Lutalin®. CLA + EFA = Lutalin® + Linseed oil + Safflower oil*.

2 *Proportions of presented fatty acids out of the total amount of fatty acids*.

3 *EPA, eicosapentaenoic acid*.

4 *DPA, docosapentaenoic acid*.

5 *DHA, docosahexaenoic acid*.

6 *Sum of n-3 fatty acids (18:3 cis-9, cis-12, cis-15; 18:4 cis-6, cis-9, cis-12, cis-15; 20:3 cis-11, cis-14, cis-17; 20:5 cis-5, cis-8, cis-11, cis-14, cis-17; 22:5 cis-7, cis-10, cis-13, cis-16, cis-19; 22:6 cis-4, cis-7, cis-10, cis-13, cis-16, cis-19)*.

7 *Sum of n-6 fatty acids (18:2 cis-9, cis-12; 18:3 cis-6, cis-9, cis-12; 20:2 cis-11, cis-14; 20:3 cis-8, cis-11, cis-14; 20:4 cis-5, cis-8, cis-11, cis-14; 22:2 cis-13, cis-16; 22:4 cis-7, cis-10, cis-13, cis-16; 22:5 cis-4, cis-7, cis-10, cis-13, cis-16)*.

8 *SFA, sum of saturated fatty acids*.

9 *MUFA, sum of monounsaturated fatty acids*.

10 *PUFA, sum of polyunsaturated fatty acids*.

a, b *Different lower case superscripts mark significant differences between feeding groups and different capitalized superscripts designate significant differences between male and female calves (P < 0.05)*.

MFAS of CLA + EFA increased the proportions of α-linolenic acid, eicosapentaenoic acid, docosapentaenoic acid, and n-3 fatty acids in jejunal chyme of calves compared to calves of the other two groups (*P* < 0.05). The proportions of n-6 fatty acids and polyunsaturated fatty acids (PUFA) were increased by MFAS of CLA + EFA compared to the CON group (*P* < 0.05).

**Figure 2:**
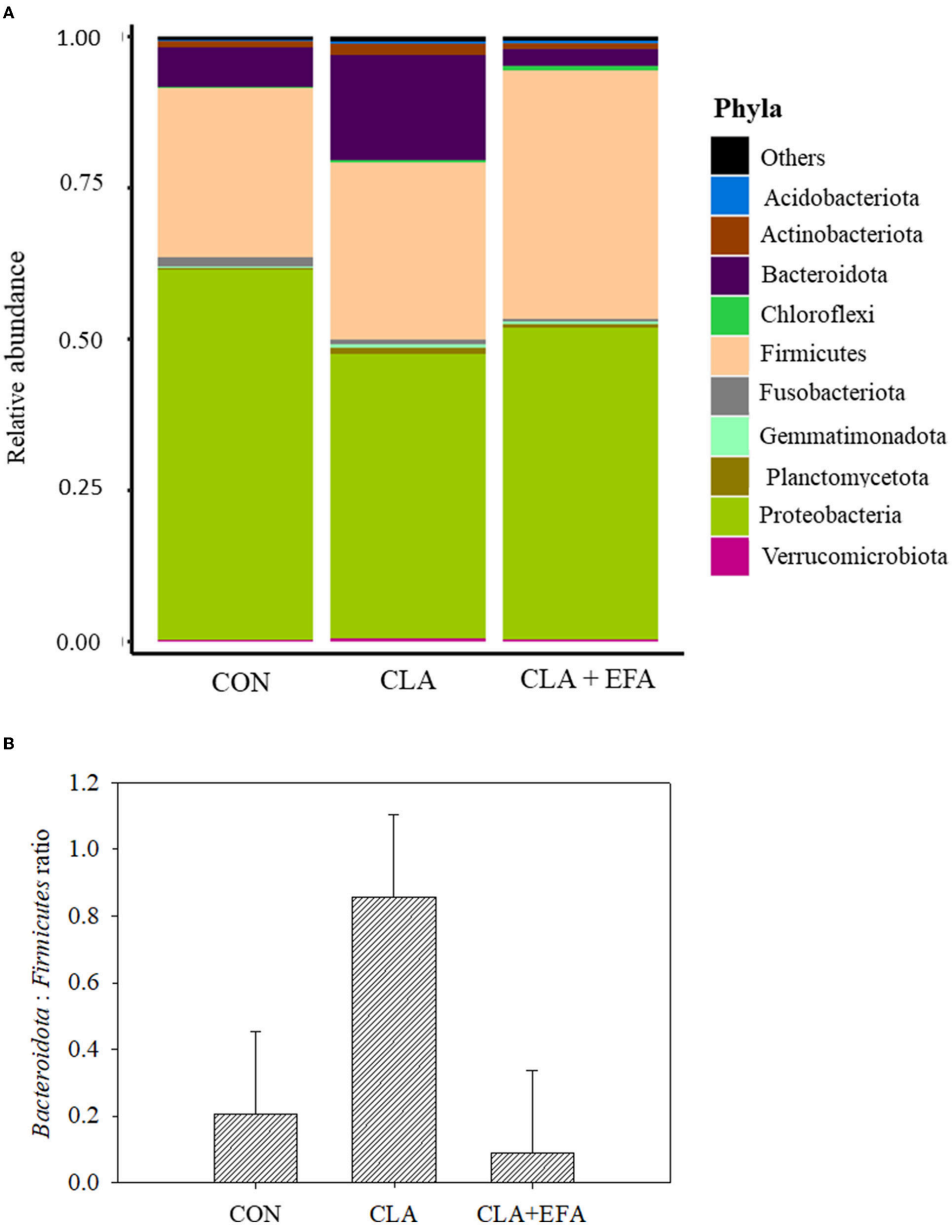
**(A)** Relative abundance of phyla **(A)** and Bacteridota: Firmicutes ratio **(B)** depending on maternal fatty acid supplementation (CLA, conjugated linoleic acid = Lutalin®. CLA + EFA = Lutalin® + linseed oil + safflower oil) in the jejunal content of 5-day-old calves (means; *n* = 3).

In all groups, *Proteobacteria* (53.4%) showed the highest relative abundance in the jejunal content of calves followed by *Firmicutes* (32.9%) and *Bacteroidota* (8.9%) [Figure 2A; http://www.ncbi.nlm.nih.gov/bioproject/795056]. The relative abundance of mentioned phyla and the *Bacteroidota*:*Firmicutes* ratio did not differ significantly between groups (Supplementary Table 1 and Figure 2B). However, the relative abundance of bacteria from the class *Diplorickettsiales* belonging to *Proteobacteria* was decreased in CLA + EFA calves (relative abundance = 0.001) compared to the CON (relative abundance = 0.120) and to the CLA (relative abundance = 0.115) calves (both *P* < 0.05). CLA calves showed a lower abundance of *Enterobacterales* (*Proteobacteria*) (relative abundance = 4.22) compared to CON calves (relative abundance = 45.00; *P* = 0.001). The abundance of bacteria from the classes *Veillonellales-Selenomonadales* and *RF39*, both belonging to *Firmicutes* were decreased in CLA + EFA (relative abundance *Veillonellales-Selenomonadales* = 0.02; *RF39* = 0.002) calves compared to the CON calves (relative abundance *Veillonellales-Selenomonadales* = 7.80; *RF39* = 0.09; *P* < 0.05). In addition, *Bacteroidales* (*Bacteroidota*) were decreased in CLA + EFA calves (relative abundance = 1.79) compared to the CLA calves (relative abundance = 15.42; *P* < 0.05). The relative abundance of *Cyanobacteria* and *Euryarchaeota* was decreased in CLA + EFA calves (relative abundance *Cyanobacteria* = 0.001; *Euryarchaeota* = 0.002) compared to CON (relative abundance *Cyanobacteria* = 0.041; *Euryarchaeota* = 0.067) and to CLA (relative abundance *Cyanobacteria* = 0.034; *Euryarchaeota* = 0.116) calves (both *P* < 0.05). *Euryarchaeota*, which differed significantly between these groups, belonged to the class of the *Methanobacteriales* (relative abundance CON = 0.068; CLA = 0.118; CLA + EFA = 0.002; CLA + EFA vs. CON and CLA, P < 0.05, respectively) and the order of the *Methanobacteria* (relative abundance CON = 0.067; CLA = 0.117; CLA + EFA = 0.002; CLA + EFA vs. CON and CLA, *P* < 0.05, respectively). In contrast, the relative abundance of *Chloroflexi* bacteria was increased in the CLA + EFA group (relative abundance = 0.74) compared to the CON (relative abundance = 0.15) and CLA (relative abundance = 0.37) group (*P* < 0.05). These bacteria included especially the class of the *SBR1031*.

## Discussion

The early intestinal microbiota plays a crucial role in direct and long-term health of neonates (10–12). The maternal colostrum and contained fatty acids might be also an important factor in the development of the microbiota (7). In turn, CLAs were demonstrated to influence both the fatty acid composition in colostrum or milk of dairy cows and the intestinal microbiota of mammals (4–6, 8, 9). The present study investigated the effects of a maternal CLA supplementation with or without an additional EFA supply on physico-chemical characteristics of the intestinal content and the microbial composition in 5-day-old calves.

According to studies of Song et al. (21), acetate was the dominating VFA in the small intestine of neonatal calves. In contrast to the mentioned study, propionate was not detected, which might be based on the different sampling locations. In mice, trans-10, cis-12 CLA increased the levels of acetate, propionate, and iso-butyrate in cecum, which might indicate an increased microbial fermentation (9). In the present study, there was no clear effect of CLAs on VFA, which might be based on the low animal number. Interestingly, only in control calves butyric acid was detected in the jejunum. Marques et al. (9) found no effect on n-butyrate in the murine intestinal content but their investigations were limited on cecal content. Butyric acid is known for its beneficial effects on intestinal gluconeogenesis and anti-inflammatory effects and influences the lipid metabolism of the host (8).

The proportions of unsaturated fatty acids measured in the intestinal content reflected mainly the modulations of fatty acids in colostrum of supplemented dams and blood of calves as demonstrated in a companion paper by Liermann et al. (22). Although it was not significant, CLA was numerically increased in the intestine of groups with CLA supply.

Corresponding to studies of Song et al. (21), *Proteobacteria* and *Firmicutes* dominated the small intestine of neonatal calves, and the most abundant Archaea were *Euryarchaeota*. Marques et al. (9) reported reduced proportions of *Firmicutes* and increased proportions of *Bacteroidota* in murine cecum by supplementing trans-10, cis-12 CLA. In the present study, these phyla were not significantly affected; however, *Veillonellales-Selenomonadales* and *RF39*, both belonging to the *Firmicutes* were affected by the maternal CLA + EFA supplementation. Because the CLA group showed no significant difference in these classes, the effect of the CLA + EFA group might be mainly based on the influence of EFA. However, these results should be considered with care because the abundance of *Veillonellales-Selenomonadales* and *RF39* showed a considerable variation among the calves. The differences between the CLA and the CLA + EFA calves become clearer with regard to the differences in *Bacteroidales* belonging to the *Bacteroidota*, which were decreased in CLA + EFA compared to CLA calves. Although Marques et al. (9) only used trans-10, cis-12 CLA, they found an increasing effect of CLAs on *Bacteroidota* in cecum of mice.

The more pronounced effects of the maternal supplementation of CLA + EFA on the microbial composition might be based on the higher concentration of unsaturated fatty acids in the intestinal content. As summarized by Costantini et al. (23) and Machate et al. (9), especially, PUFA have far-reaching influences on the gut microbiota including various classes and families of *Firmicutes, Bacteroidota*, and *Proteobacteria*. Results of Robertson et al. (10) in mice studies indicated an important impact of n-3 PUFA in milk on the gut microbiota of the offspring.

The biological importance of the presented modulations in the intestinal microbiota has to be proven in further studies with a larger number of animals. However, in previous studies, relationships between hepatic metabolism and metabolic disorders and modulations in gut microbiota were observed (8, 9, 24). In general, it is also known that the intestinal microbiota plays a key role in the development of the gastro-intestinal tract and the intestinal immune system (12). Furthermore, fermentation products of the microbial community such as short-chain fatty acids have a crucial impact on host homeostasis and disease (8). Prolonged expansion of *Enterobacteriaceae* belonging to the class of *Enterobacterales*, seemed to be significantly reduced by the MFAS of CLA (family data not shown), was shown to play a crucial role in the development of diarrhea in calves (25). Therefore, it might be assumed that CLA could have beneficial effects in the avoidance of diarrhea. *Euryarchaeota* belong to the Methanogens, which are responsible for ruminal enteric methane synthesis and are discussed to be involved in glycolysis and the host immune response (21, 26, 27). Little is known about the function of *Chloroflexi* because usually this phylum can be found in aquatic or terrestrial habitats (28). However, this phylum was also detected in the mammalian gastrointestinal tract in previous studies (28, 29). Human studies of Campbell et al. (28) give evidence that oral *Chloroflexi* show similar to environmental *Anaerolineae* an abundant carbohydrate transport and metabolism. *Cyanobacteria* were also found in the colonic mucosa of pre-weaning calves in studies of Guo et al. (30). Their abundance increased during weaning (30). In the past, especially, environmental cyanobacteria received attention because of some species producing harmful cyanotoxins (31–33). The function and biological importance of intestinal species belonging to *Cyanobacteria* have to be investigated in further studies, but the abundance of this phylum was low compared to the other phyla. Finally, especially, Robertson et al. (10) indicated that modulations of the microbiota in the neonatal period by fatty acid supply have a persistent effect on the offspring microbiota in a later stage of life.

## Conclusion

In conclusion, the present study gives evidence that the maternal fatty acid supply alters the fatty acid concentrations in the intestinal content due to their ability to modulate the colostral fatty acid composition of dams. The changes in the chemical characteristics of the intestinal content might contribute to changes in the microbial milieu of the intestine. Further studies are needed to support these preliminary findings and to investigate the direct and long-lasting biological relevance of these alterations.

## Data Availability Statement

The datasets presented in this study can be found in online repositories. The names of the repository/repositories and accession number(s) can be found at: NCBI SRA database, accession number PRJNA795056.

## Ethics Statement

The animal study was reviews and approved by the Landesamt für Landwirtschaft, Lebensmittelsicherheit, und Fischerei Mecklenburg-Vorpommern, Rostock (registration number 7221.3-1-052/15).

## Author Contributions

WL and HH: conceptualization. WL, DD, HR, and NT: methodology. WL and KW: investigation. AT and HH: resources and funding acquisition. WL: writing—original draft preparation. HR and HH: writing—review and editing. HH: supervision and project administration. All authors contributed to the article and approved the submitted version.

## Funding

This work was supported by BASF SE (Ludwigshafen, Germany). The publication of this article was funded by the Open Access Fund of the Leibniz Institute for Farm Animal Biology (FBN).

## Conflict of Interest

AT was employed by BASF SE. The remaining authors declare that the research was conducted in the absence of any commercial or financial relationships that could be construed as a potential conflict of interest.

## Publisher's Note

All claims expressed in this article are solely those of the authors and do not necessarily represent those of their affiliated organizations, or those of the publisher, the editors and the reviewers. Any product that may be evaluated in this article, or claim that may be made by its manufacturer, is not guaranteed or endorsed by the publisher.
