# Supplementary material for: Maternal Conjugated Linoleic Acid Supply in Combination With or Without Essential Fatty Acids During Late Pregnancy and Early Lactation: Investigations on Physico-Chemical Characteristics of the Jejunal Content and Jejunal Microbiota in Neonatal Calves
Source: Front Vet Sci. 2022 Mar 25;9:839860. doi: 10.3389/fvets.2022.839860 (PMC8994029; doi:10.3389/fvets.2022.839860)
Supplement: Supplementary file 1 [file Table_1.DOCX]

SUPPLEMENTAL TABLE 1 | Relative abundance (%) of detected phyla, classes and orders in jejunal intestine differing between calves with different maternal fatty acid supplementation (MFAS)^1^

| **Species** | **MFAS** | | |  | **P values^2^** | | |
| --- | --- | --- | --- | --- | --- | --- | --- |
|  | **CON**  **(n = 3)** | **CLA**  **(n = 3)** | **CLA+EFA**  **(n = 3)** |  | **CON vs.**  **CLA** | **CON vs. CLA+EFA** | **CLA vs. CLA+EFA** |
| Phylum |  |  |  |  |  |  |  |
| *Cloroflexi* | 0.153 | 0.370 | 0.736 |  | 0.875 | < 0.001 | 0.019 |
| *Cyanobacteria* | 0.041 | 0.034 | 0.001 |  | 0.875 | < 0.001 | < 0.001 |
| *Euryarchaeota* | 0.067 | 0.116 | 0.002 |  | 0.875 | 0.001 | 0.001 |
|  |  |  |  |  |  |  |  |
| Class |  |  |  |  |  |  |  |
| *Bacteroidales* | 5.620 | 15.424 | 1.787 |  | 0.730 | 0.470 | 0.045 |
| *Gemmatales* | 0.097 | 0.182 | 0.000 |  | 0.944 | < 0.001 | < 0.001 |
| *Diplorickettsiales* | 0.120 | 0.115 | 0.001 |  | 0.963 | 0.011 | 0.015 |
| *Enterobacterales* | 46.996 | 4.216 | 17.235 |  | 0.001 | 0.317 | 0.084 |
| *Methanobacteriales* | 0.068 | 0.118 | 0.002 |  | 0.944 | 0.003 | 0.015 |
| *RF39* | 0.086 | 0.077 | 0.002 |  | 0.944 | 0.028 | 0.081 |
| *SBR1031* | 0.030 | 0.041 | 0.394 |  | 0.970 | 0.025 | 0.025 |
| *Veillonellales-Selenomonadales* | 7.798 | 0.847 | 0.025 |  | 0.305 | < 0.001 | 0.099 |
|  |  |  |  |  |  |  |  |
| Order |  |  |  |  |  |  |  |
| *Kiritimatiellae* | 0.008 | 0.078 | 0.001 |  | 0.731 | 0.143 | 0.014 |
| *Methanobacteria* | 0.068 | 0.117 | 0.002 |  | 0.991 | < 0.001 | < 0.001 |
| *Negativicutes* | 7.761 | 0.841 | 0.033 |  | 0.603 | 0.003 | 0.128 |
| *Vampirivibrionia* | 0.032 | 0.031 | 0.000 |  | 0.992 | 0.001 | 0.005 |
|  |  |  |  |  |  |  |  |

^1^ CON, control group = coconut oil. CLA, conjugated linoleic acid = Lutalin^®^. CLA + EFA = Lutalin^®^ + linseed oil + safflower oil.

^2^ Differences were considered significant at a Benjamini-Hochberg adjusted P-value of P < 0.05.
